# The initial break-up of Pangæa elicited by Late Palæozoic deglaciation

**DOI:** 10.1038/srep31442

**Published:** 2016-08-11

**Authors:** Meng-Wan Yeh, J. Gregory Shellnutt

**Affiliations:** 1 Department of Earth Sciences, National Taiwan Normal University, 88 Tingzhou Road Section 4, Taipei 116, Taiwan; 2 Center for General Education, National Taiwan Normal University, 162 Heping East Road Section 1, Taipei 106, Taiwan

## Abstract

The break-up of Pangæa was principally facilitated by tensional plate stress acting on pre-existing suture zones. The rifting of Pangæa began during the Early Permian along the southern Tethys margin and produced the lenticular-shaped continent known as Cimmeria. A mantle-plume model is ascribed to explain the rift-related volcanism but the NW-SE oriented Cimmerian rifts do not correlate well with pre-existing suture zones or ‘structural heterogeneities’ but appear to have a pertinent spatial and temporal association with Late Palæozoic glacial-interglacial cycles. Mantle potential temperature estimates of Cimmerian rift-related basalts (1410 °C ± 50 °C) are similar to ambient mantle conditions rather than an active mantle-plume rift as previously suggested. Moreover, we find that the distribution of glacial deposits shows significant temporal and spatial concurrence between the glacial retreat margins and rifting sites. We conclude that the location and timing of Cimmerian rifting resulted from the exploitation of structural heterogeneities within the crust that formed due to repeated glacial-interglacial cycles during the Late Palæozoic. Such effects of continental deglaciation helped to create the lenticular shape of Cimmeria and Neotethys Ocean suggesting that, in some instances, climate change may directly influence the location of rifting.

Crustal thinning, narrow rifting characterized by localization of strain through large boundary fault activation, and basin subsidence are the signature features of the early stages of continental break-up^1^,^2^. Transient excess magmatic upwelling and emplacement covering the inboard portions of continental rift margins follow the early stages of rifting^2^,^3^,^4^. Continent break-up/rifting requires tremendous amount of energy. In order to conserve energy, initial rifting sites within continental crust are often spatially associated with pre-existing ‘structural heterogeneities’ such as suture zones related to ancient orogenic boundaries or regions of the crust with differential thickness^2^,^5^,^6^,^7^. A given ‘structural heterogeneity’ may be exploited by either a mantle-plume (active extension) or a regional stress field (passive extension) associated with plate boundary forces (slab-pull) that becomes a focal point for flood basalt volcanism before developing into an ocean basin^8^. Moreover, icosahedral structures with triple-junction configuration form quasi-hexagonal fractures that minimize the total boundary length and produce polyhedral plate configurations, thus minimizing the energy, area, work and stress required to brittely break plates^9^,^10^.

The amalgamation and break-up of Pangæa exemplifies the secular nature of global tectonic processes as plate stresses transitioned from compressional to extensional during the Late Carboniferous to Early Jurassic^6^,^10^,^11^,^12^. There are a number tectonomagmatic models proposed to explain the break-up of Pangæa such as: post-orogenic collapse^13^, dispersal over a mantle-super plume^14^, passive rifting and self-subduction of a super-plate^11^,^15^. However, in many cases, rifting and magmatism were initiated at old suture zones that formed during the amalgamation stage. Some of the hypotheses provide a partial explanation for the break-up of the Gondwana portion of Pangæa specifically during the Jurassic to Cretaceous but do not adequately address an earlier period of rifting^5^,^11^.

The break-up of Gondwana began during the Early Permian along a ~13,000 km long NW-SE oriented continental rift just as the Late Palæozoic continental ice sheet retreated^16^,^17^,^18^. The rifting of terranes from the Tethyan margin of Gondwana produced the ribbon-like continent Cimmeria and the Neotethys Ocean. The rifting of Cimmeria and accompanying magmatism are thought to be related to a regional-scale mantle-plume^19^,^20^,^21^. However, geochemical and structural studies of the Panjal Traps (Kashmir), the single largest contiguous outcropping of Early Permian flood basalts associated with Cimmerian rifting, suggest they are related to passive extension that propagated eastward^22^,^23^,^24^. In contrast, Early Permian basaltic rocks in Oman (west) and Tibet (east) are each thought to be derived by a mantle-plume and that the Panjal Traps are either an eastern or western extension of rift propagation^19^,^20^,^21^,^25^. There are a few discrepancies with ascribing a mantle-plume model to the formation of Cimmeria. For example: 1) plate separation did not occur at or near suture zones that would later be exploited by mantle-plumes during the Mesozoic break-up of East Gondwana (Fig. 1a)^26^, 2) a clearly defined aulacogen associated with the Cimmerian rifts, a feature typical of Mesozoic Pangæan rifts, has not been identified, 3) the Cimmerian rift boundary follows the stress tessellation instead of following the fracture tessellation that produces polyhedral plates (Fig. 1f), and 4) there are no ultramafic volcanic rocks or large radial mafic dyke swarms^2^,^26^,^27^,^28^ associated with rifting.

A lenticular ribbon shape continent is not unique to Cimmeria as rifted peri-Gondwana terranes (Avalonia, Hunia) had ribbon-shapes during the opening of Rheic Ocean^26^ as well as the Galatian super terrane during the development of the Palæeotethys. The peri-Gondwana terranes mostly originated as part of collisional island arc systems. For example, Avalonia and Hunia belonged to the Cadomian arc system^26^,^29^ whereas the Galatian super terrane was a portion of the Variscan orogeny^30^ and Hanseatic arc^31^. It is likely that inherited structural heterogeneities within the peri-Gondwana terranes were reactivated during continental break up.

To better understand the tectonomagmatic development of Cimmeria, we investigate the thermal regime of the basaltic rocks associated with rifting and the structural controls that led to the formation of its lenticular shape. Anomalously hot (>1550 °C) or ambient (1350 ± 50 °C) mantle potential temperatures (*T*_P_) required to generate the primary melt composition of basalt from within-plate or extensional settings can be calculated from their bulk composition. Thus the mafic rocks from Oman, Kashmir and Tibet can be used to deduce the regional thermal conditions during Early Permian rifting^32^,^33^. Furthermore, a detailed geological examination of the rifted margin can help elucidate the boundary conditions that influenced the rift orientation and created the lenticular shape of Cimmeria.

## Mantle potential temperature estimates of the Cimmerian rift basalts

The Hawasina, Panjal, Arbor, Nar-Tsum, Bhote Kosi, Selong, Mojiang volcanic groups, Qiangtang mafic dykes and the Garze Ophiolite are amongst the basaltic rocks within the Tethyan terranes of Oman, Pakistan, India and China^19^,^23^,^34^,^35^,^36^,^37^,^38^,^39^. Not all of the volcanic and intrusive units are well studied but they are interpreted to be related to the rifting of Cimmeria and formation of the Neotethys Ocean during the Early Permian (300 Ma to 280 Ma).

Basalts that fractionated plagioclase, pyroxene or both are not suitable for primary melt and *T*_P_ calculations because the initial adiabatic temperature-pressure melting path of basalt is similar to the olivine liquidus^32^. Consequently only rocks that fractionated olivine can produce meaningful results^36^. We calculated the anhydrous accumulated fractional melting primary magma compositions of the Cimmerian rift basalts from Oman, Kashmir and Tibet at atmospheric pressure under both relatively reducing (Fe_2_O_3_/TiO_2_ = 0.5) and relatively oxidizing conditions (Fe_2_O_3_/TiO_2_ = 1.0). The relatively reducing primary melt models produced compositions that range from high-Mg basalt to picrite (MgO = 12.1 wt% to 15.8 wt%) with corresponding eruption (T) and mantle potential (*T*_P_) temperatures between 1260 °C and 1360 °C and 1360 °C and 1460 °C respectively (Fig. 2a). The relatively oxidizing primary melt models are broadly similar to the reducing models in composition but yielded T and *T*_P_ values 20 °C to 50 °C lower for some samples (Dataset S2). Irrespective of the relative oxidation state used in the models, the results show that the temperature of the mantle that produced the primary magmas of the basalts was not anomalously hot but rather similar to ambient mantle conditions (1300 °C to 1400 °C).

Mantle-plumes are thought to have a diameter of ~2500 km as they flatten-out near the top of the mantle^27^. Therefore it is unlikely a single mantle-plume could be responsible for volcanism along a ~13 000 km rift zone. Figure 2b shows the range of ε_Nd_(t) values of Cimmerian rift-related basalts. Some of the isotopic variability in the basalts is undoubtedly related to crustal contamination but there is no systematic spatial variation. The Oman, Qiangtang and Mojiang basaltic rocks tend to have more depleted ε_Nd_(t) signatures than the Panjal and Tibet basalts implying local mantle sources (sub-lithospheric vs. lithospheric) were likely responsible for their genesis. Moreover, the *T*_P_ values are not sufficiently different to distinguish a regional thermal trend that would be indicative of a mantle-plume regime (hotter core, cooler margin)^40^. The implication is that the Early Permian basalts were not generated within a mantle-plume thermal regime but are more similar to conditions identified at passive rifts and other (i.e. Central Atlantic Magmatic Province, Karoo LIP, North Atlantic LIP) non-plume large igneous provinces^41^,^42^,^43^,^44^. Therefore it is highly probable that the Cimmerian rift basalts formed due to passive extension related to plate stress rather than a mantle-plume and that the orientation of the rift basins were structurally controlled by heterogeneities within the crust.

## Formation of the Early Permian rifts

**Figure 1:**
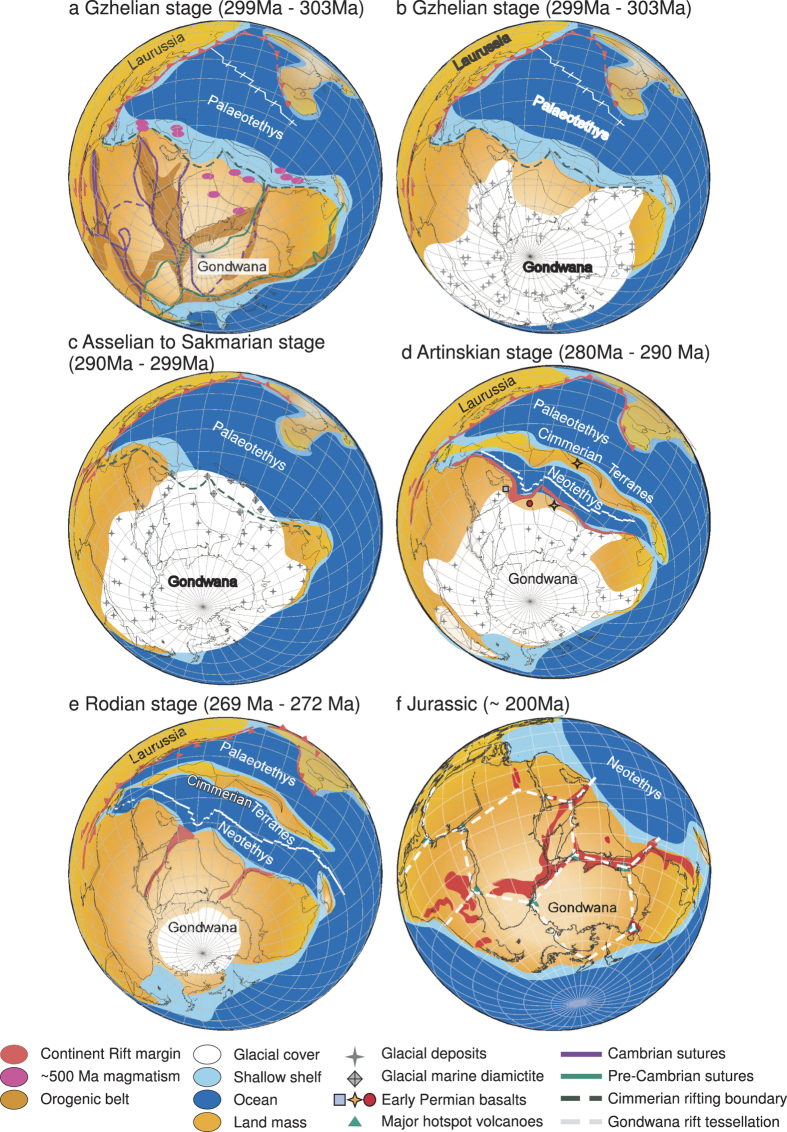
Early Permian paleo-geographic map reconstructions of Pangæa via open-source software GPlates 1.5 and the database provided within: http://www.gplates.org/index.html. Please refer to “method” section for detailed settings and list of references. Further geological features such as: pre-existing suture^15^, orogeny belts, and glacial deposits (Dataset S1) are superimposed on the paleo-geographic locations. (**a**) Gondwana during the Gzhelian stage (299 Ma–303 Ma) showing the distribution of pre-existing Precambrian (thick green lines) and Cambrian (thick purple lines) suture zones, and orogenic belts (dark brown region). The pink region marks the distribution of Pan-African (ca. 500 Ma) magmatism^45^. The continent rift margin that separated the Cimmerian terranes is marked by dark green dashed line that does not follow pre-existing suture zones but cuts through Pan-African (500 Ma) magmatic rocks. (**b**) Glacial cover before the Gzhelian stage (299 Ma–303 Ma) indicated by the distribution of glacial deposits on land (grey cross, Dataset S1). (**c**) Maximum glacial extent through Asselian to Sakmarian stage (290 Ma–299 Ma) indicated by the distribution of glacial deposits on land (grey cross) and glacial marine diamictite (grey diamond). (**d**) The beginning of Neotethys rifting and breakoff of Cimmerian terranes around the Artinskian stage (280 Ma–290 Ma). The blue square (Oman), red circle (Kashmir), and orange cross (Tibet) mark the location of Early Permian basalts. The red region marks the continent rift margin. Both temporal and geographical correlation can be observed for the glacial retreat, continent rift margin, and the distribution of Early Permian basalts. (**e**) Continued spreading of the Neotethys Ocean and further rifting between Africa, India and Australia (red region) during the Rodian stage (269 Ma–272 Ma). (**f**) Fracture tessellation defined by major rift margins around Jurassic time (~200 Ma). Such configuration is similar to an icosahedron arrangement composed of numerous pentagons with hexagons as marked by the white dashed lines^10^.

**Figure 2:**
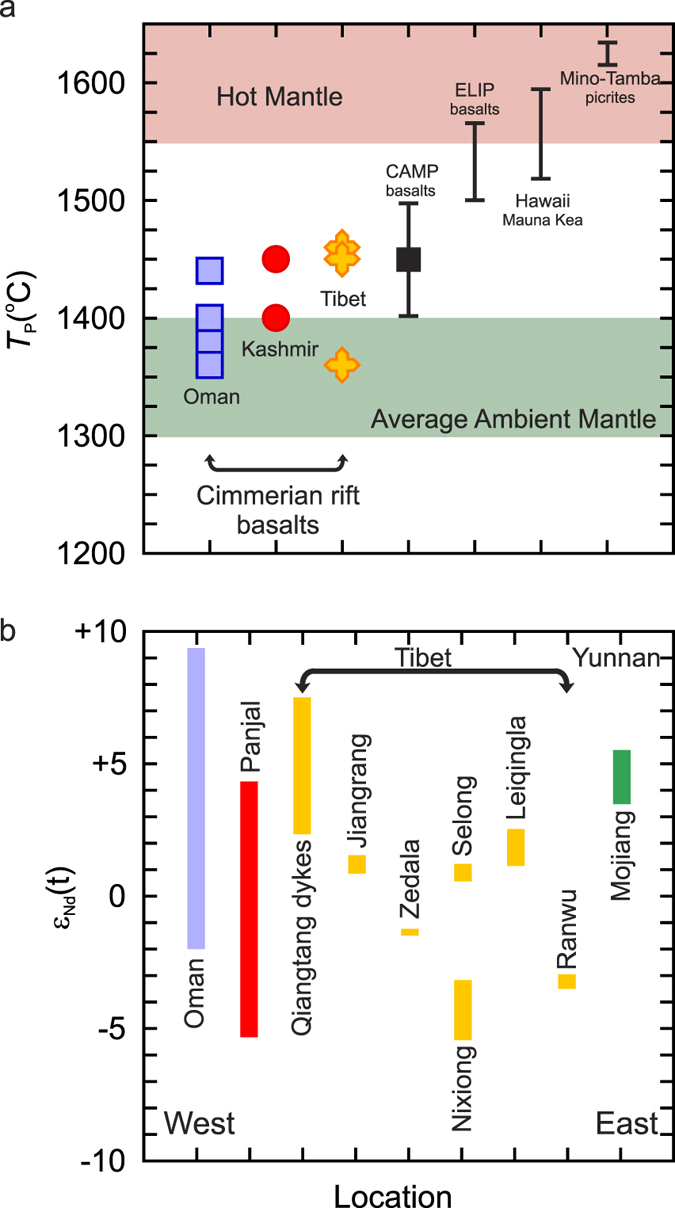
Mantle potential temperature (*T*_P_) estimates of the Early Permian Cimmerian basalts and variability of Nd isotopes of Cimmerian basalts. (**a**) *T*_P_ estimates of the Cimmerian rift basalts in comparison with results from the Central Atlantic Magmatic Province, Emeishan large igneous province (China), Hawaii (Mauna Kea) and Mino-Tamba (Japan). Calculations parameters are summarized in Dataset S2. (**b**) The variability of ε_Nd_(t) of Early to Middle Permian mafic volcanic rocks from west to east across the Himalaya^19^,^21^,^24^,^36^,^37^,^39^,^70^.

The northward directed slab pull under the E-W trending subduction zones along the northern margin of the Palæotethys is considered to be the main driving mechanism that generated the NW-SE trending ‘Cimmerian rifts’ (Fig. 1c)^11^,^12^, yet without pre-existing ‘structural heterogeneities’, no generic relationship can be established to demonstrate the occurrence criteria for rifting sites and the linear extent of rifts over thousands of kilometers. Our plate and geological reconstruction of the Early Permian Tethyan margin of Gondwana indicate that the rifting sites of Cimmeria, marked by temporal and geographical distribution of syn- to post- rifting volcanism along the southern part of the higher Himalaya, do not appear to follow pre-existing structural heterogeneities (Fig. 1a). There are a number of Cambrian Andean-type batholiths along the pre-Tethyan segment of Gondwana that could have behaved as a potential structural heterogeneity but rifting penetrated through the center of the magmatic arc instead of its boundary (Fig. 1a)^12^,^30^,^31^,^45^. Therefore, in order to resolve the controlling mechanism required to form a ribbon-shaped continent, the mechanical properties of the lithosphere and the temporal and spatial variability of the plate strength must be considered^46^. In other words, the structural weak zone likely developed prior to the occurrence of major detachment during the Late Palæozoic.

## A link between deglaciation and the formation of structural heterogeneities

The Late Palæozoic is characterized as a time of large continental and alpine glaciers that covered the Polar regions of southern Gondwana^47^,^48^. Although the northern margin of Gondwana had transformed from an Andean-type convergent margin during the Early Palæozoic to a passive rift margin during the Late Palæozoic, major rifting pulses marked by Carboniferous-Permian unconformities and hiatuses within numerous Gondwanan basins occurred during the warmer deglaciation periods^17^. The temporal coincidence between rifting and deglaciation suggests there is a possible link between the two.

**Figure 3:**
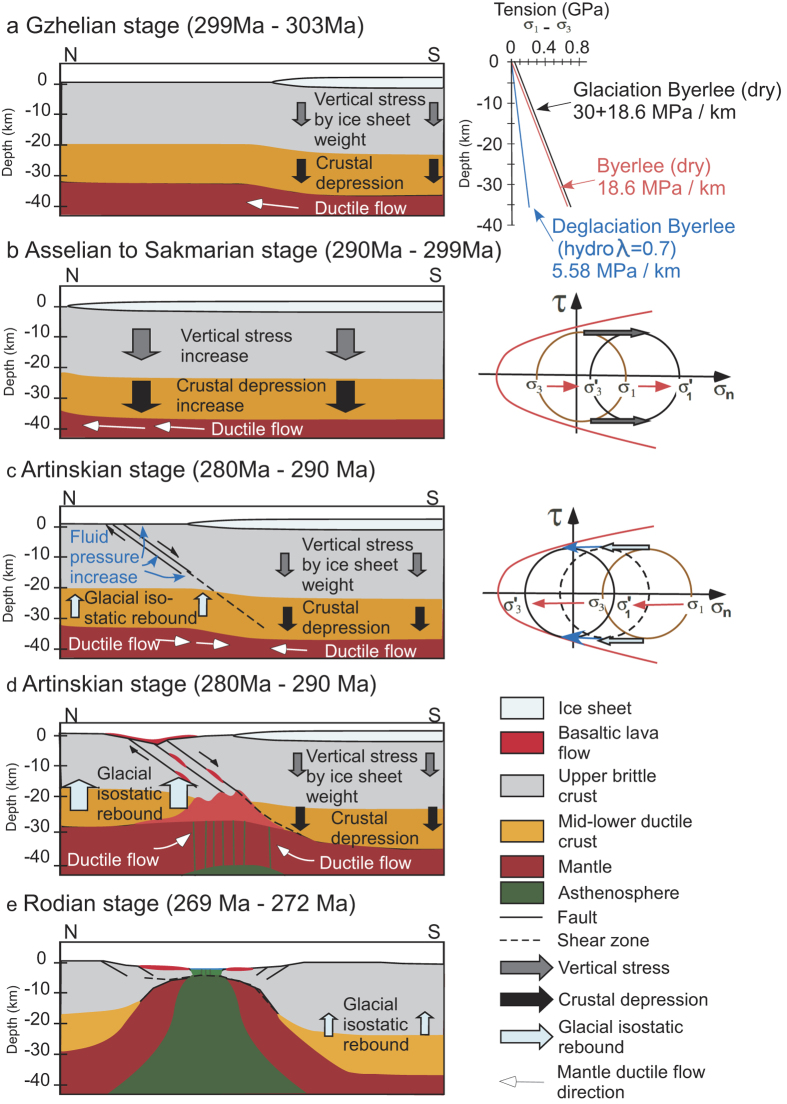
Schematic diagrams illustrating crustal evolution and the development of rifting under passive extensional tectonic setting. N-S trending cross sections showing the relative thickness of the brittle crust (grey), ductile crust (orange) and mantle (brown). The thickness of ice sheet (pale blue) is assumed to be 3–4 km that induces ~1 km of lithospheric depression^53^. (**a)** Hypothetical crustal profile with ice cover during the Gzhelian stage (299 Ma–303 Ma). The region under ice cover is affected by the vertical stress (grey arrow) from the weight of ice sheet. The estimated brittle strength for the upper crust is (ơ_1_ − ơ_3_) equal to 30 + 18.6 MPa km^−1^ and would induce crustal depression (black arrow) and ductile flow (white arrow) of the mantle away from the center of the ice sheet^50^. (**b)** Northward advancement of ice sheet around Asselian to Sakmarian stage (290 Ma–299 Ma) further depressed the crust and increased mantle flow. Brittle deformation is suppressed as the increase of vertical stress moved the stress circle (brown - original state; black – finite state) further away from the Mohr failure envelop (red line). (**c)** Early stage of glacial retreat around the Artinskian stage (280 Ma–290 Ma). The deglaciated region experiences isostatic rebound (pale blue arrow) with estimated brittle strength for the upper crust to be (ơ_1_ − ơ_3_) equal to 18.6 MPa km^−1^ 50. The melted glacier water penetrates the upper crust and increases the fluid pressure (blue arrow) and reduces the brittle strength significantly (ơ_1_ − ơ_3_ = 5.58 MPa km^−1^)^55^. The combination of isostatic rebound (brown to dashed black circle) and increased fluid pressure (dashed black to black circle) moves the stress circle towards the Mohr failure envelop and triggers brittle failure and fault swarm development. (**d)** The stress heterogeneity between deglaciated and ice covered regions further weaken the crust. Mantle backflow and crustal rebound induced decompressional melting. Mantle-derived melts upwelled along pre-developed fault zones forming regional flood basalts around the later Artinskian stage (280 Ma–290 Ma). (**e**) Further extension of the crust and ascending of asthenosphere (green) around Rodian stage (269 Ma–272 Ma) completed the break off of Cimmerian terranes.

Incorporating surface processes such as glaciation and weathering to internal lithosphere dynamics has long been explored since the establishment of the glacio-isostasy concept^49^. According to Byerlee’s Law, the strength of brittle continental crust is determined by a function of pressure-depth rather than rock types or structures making brittlely deformed crust behave as a mechanically uniform media^50^. Therefore, large boundary faults can propagate over large distances at depth regardless of the inherited geological features^46^. However, in order to initiate brittle failure, typical continental crust requires stress roughly twice the confining pressure. Thus, thicker overburden would lead to larger confined pressure and vertical stress, making brittle failure less likely to occur and explains why large continental ice sheets behave as a protective shield, and tend to suppress tectonic activity^51^,^52^. Seismo-tectonic activity and large boundary fault movements occur at the deglaciation front and after glaciation due to glacial isostatic adjustment (GIA) and are widely recognized along both active continental margins and anorogenic settings^53^,^54^. Numerical modeling, based upon Fennoscandia, demonstrated a significant decrease of both normal and shear stress and fault stability for regions with an increase of glacial unloading^53^. Rheological laws indicate the yield strength of the dry upper crust is governed by Byerlee’s Law and are three times weaker under an extensional regime than under a compressional regime (Fig. 3a)^50^. The maximum brittle strength relative to depth of the passive rifted Cimmerian upper crust without the influence of glacial loading can be estimated (ơ_1_ − ơ_3_) to be 18.6 MPa km^−1^ (Fig. 3a)^50^. Based on numerical modeling results^53^, glacial loading would increase the vertical stress to ~30 MPa and consequently depress the lithosphere by ~1 km. Glacial unloading not only reduces the vertical loading stress but the horizontal rebound stress is also reduced up to 7 MPa approximately 10 ka after deglaciation regardless of the background tectonic stress. The 30 MPa vertical loading stress does not significantly increase the brittle strength of the upper crust. However, large differential stress environments can be expected for the deglaciation front as the covered region experiences higher normal and horizontal shear stresses (ơ_1_ − ơ_3_ = 30 + 18.6 MPa km^−1^, Fig. 3)^50^ whereas the retreated region experiences strong postglacial rebound stress (lower normal and horizontal shear stresses). Repeated advance and retreat cycles of major ice sheets can cause major changes in vertical load, fluid pressures and crustal strain that can trigger elastic flexing of the lithosphere and viscous flow in the mantle, hence generate focused regions of structural heterogeneities for tectonic stress to act upon (Fig. 3)^53^,^54^. By taking the increased hydrostatic pore pressure (λ = 0.7) due to deglaciation into account, the estimated brittle strength of the upper crust after deglaciation is (ơ_1_ − ơ_3_) 5.58 MPa km^−1^ (Fig. 3)^55^. Furthermore, glacier melt water may fill pore spaces and newly developed fractures that further weaken the upper crust and allows for massive concentrated fault swarm development. Mantle backflow associated with glacial isostatic rebound may induce decompressional melting and thus mantle-derived melts percolate along previously developed fault zones and produce the regional flood basalts as terranes rift away (Fig. 3).

Although the duration, extent, style and demise are debated, the presence of marine glacial deposits around the Kashmir Valley and Salt Range (Pakistan), Southern Qiangtang (Tibet), and Australia is evidence that this region experienced Late Palæozoic glaciation during the latest Carboniferous to earliest Permian^17^,^56^. Our reconstruction based upon Asselian and Sakmarian glacial deposits show not only temporal, but geographical coincidence of glacial retreat margins and Cimmerian rifting sites (Fig. 1d). Given the effects of glacial loading on the lithosphere, we suggest that the Southern hemisphere ice sheet may have significantly contributed to the development of structural heterogeneities along an NW-SE trend of Tethyan Gondwana. The formation of structural heterogeneities near the margin of Tethyan Gondawana likely enabled the generation of a linear passive rift margin that extended thousands of kilometers and acted as preferred rifting sites during the opening of the Neotethys Ocean (Fig. 1).

## Conclusions

There is a spatial and temporal association between glacial retreat and rifting of Cimmerian terranes from Gondwana during the Late Palæozoic. Ambient mantle potential temperatures estimates of Early Permian Cimmerian rift-related basalts from Oman, Kashmir and Tibet are supportive of an archetypical passive continental rift rather than an active mantle-plume rift with anomalously hot mantle (*T*_P_ > 1550 °C). Although no inherent structural heterogeneity zones or icosahedral tessellation boundaries were present for rift margins to act upon, it is likely that a large differential stress environment, strong postglacial rebound stress, and the shielding effect of the ice sheet created regions of structural heterogeneities for a later extensional tectonic stress to exploit. Therefore, it is very likely that the eruption of flood basalts, rifting of Cimmeria and the formation of the Neotethys Ocean are directly related to deglaciation of the Late Palæozoic ice sheet. Such a relationship between environmental change and supercontinent break-up may be unique to the Late Palæozoic.

## Methods

### Plate reconstruction

GPlates 1.5 software and the sample source data provided: http://www.earthbyte.org/Resources/earthbyte_gplates_1.5_data_sources.html is utilized to reconstruct paleogeography of the breakup of Gondwana and Cimmerian terrane from 300 Ma to 270 Ma. Present-day configuration for major continent, glacial deposits, and flood basalt distribution is utilized. Present-day vector geometry and raster data is attached on a globe surface. Plate tectonic rotation models are used to restore the paleo-spatial arrangement according to poles of rotation for various plates through time. The poles of rotation for each tectonic plate passes through the earth center with an angle that changes over geological time^26^,^57^,^58^,^59^,^60^,^61^,^62^,^63^. The sample source data rotation file describes the motions of the continents and oceans are based on data obtained prior to 2012^64^. Additional poles of rotation are converted by compiled paleo-magnetic poles for different regions through time^59^,^65^,^66^,^67^. Plates rotate rigidly across the globe surface with Africa as the center for relative motion. The commonly accepted versions of Gondwana configuration are adopted with updated paleo-positions of Lhasa, Sibumasu, and the estimated space for greater India^44^,^59^,^65^,^67^,^68^,^69^. Afghanistan moved in pair with Iran after 290 Ma^12^,^16^,^19^,^66^, Sibumasu moved in pair with Qiantang after 289 Ma^67^,^70^,^71^,^72^. The distribution of Permo-Carboniferous glacial deposits are reconstructed via tracing the stratigraphic columns within major basins of the Gondwana terrane that have recorded Pangeo Megasequence (PMS)^17^,^56^. Local stratigraphic columns^73^,^74^,^75^,^76^,^77^,^78^,^79^ with glacial marine diamictites^56^ and reconstructed palaeomaps^80^,^81^,^82^,^83^,^84^,^85^ are also utilized for identification of glacial deposits through time. Supplementary Dataset S1 lists the detailed description of glacial deposits compiled from references^17^,^56^,^73^,^74^,^75^,^76^,^77^,^78^,^79^,^80^,^81^,^82^,^83^,^84^,^85^.

### Mantle potential temperature calculations

The primary melt compositions and mantle potential temperature estimates were calculated using PRIMELT3^24^. The major elemental data of each sample was entered into PRIMELT3 and calculated using an Fe_2_O_3_/TiO_2_ ratio of 0.5 and 1.0, pressure of 1 bar, H_2_O = 0 wt% and the lowest possible FeO content. The rock compositions and accumulated fractional melting (AFM) results are reported in Dataset S2.

## Additional Information

**How to cite this article**: Yeh, M.-W. and Shellnutt, J. G. The initial break-up of Pangaea elicited by Late Palæozoic deglaciation. *Sci. Rep*. **6**, 31442; doi: 10.1038/srep31442 (2016).

## Supplementary Material

Supplementary Information

## References

[b1] Whitmarsh R. B., Manatschal G. & Minshull T. A. Evolution of magma-poor continental margins from rifting to seafloor spreading. Nature 413, 150–154 (2001). 10.1038/35093085 11557977

[b2] Buiter S. J. H. & Torsvik T. H. A review of Wilson Cycle plate margins: a role for mantle plumes in continental break-up along sutures? Gondwana Res. 26, 627–653 (2014).

[b3] Huismans R. & Beaumont C. Depth-dependent extension, two-stage breakup and cratonic underplating at rifted margins. Nature 473, 74–78 (2011). 10.1038/nature09988 21544144

[b4] Geoffroy L., Burov E. B. & Werner P. Volcanic passive margins: another way to break up continents. Sci. Rep. 5, 14828 (2015). 10.1038/srep14828 PMC4595843 26442807

[b5] Courtillot V., Jaupart C., Manighetti I., Tapponnier P. & Besse J. On causal links between flood basalts and continental breakup. Earth Planet. Sc. Lett. 166, 177–195 (1999).

[b6] Keppie D. F. How the closure of paleo-Tethys and Tethys oceans controlled the early breakup of Pangæa. Geology 43, 335–338 (2015).

[b7] Vauchez A., Barruol V. & Tommasi A. Why do continents break-up parallel to ancient orogenic belts. Terra Nova 9, 62–66 (1997).

[b8] Buiter S. Geodynamics: how plumes help to break plates. Nature 513, 36–37 (2014). 10.1038/513036a 25186893

[b9] Anderson D. L. How many plates? Geology 30, 411–414 (2002).

[b10] Sears J. W. Lithospheric control of Gondwana breakup: Implications of a trans-Gondwana icosahedral fracture system. In: Foulger G. R. & Jurdy D. M. eds Plates, Plumes, and Planetary Processes. Geological Society of America Special Paper 430, 593–601 (2007).

[b11] Gutierrez-Alonso G. . Self-subduction of the Pangæan global plate. Nature Geosci. 1, 549–553 (2008).

[b12] Stampfli G. M., Marcoux J. & Baud A. Tethyan margins in space and time. Palaeogeogr. Palaeocl. 87, 373–410 (1991).

[b13] Henk A. Gravitational orogenic collapse versus plate boundary stresses: A numerical modelling approach to the Permo-Carboniferous evolution of Central Europe. Geol. Rundschau 86, 39–55 (1997).

[b14] Storey B. C. The role of mantle plumes in continental breakup: case histories from Gondwanaland. Nature 377, 301–308 (1995).

[b15] Stampfli G. M. & Borel G. D. A plate tectonic model for the Paleozoic and Mesozoic constrained by dynamic plate boundaries and restored synthetic oceanic isochrons. Earth Planet. Sc. Lett. 196, 17–33 (2002).

[b16] Angiolini L. . From rift to drift in South Pamir (Tajikistan): Permian evolution of a Cimmerian terrane. J. Asian Earth Sci. 102, 146–169 (2015).

[b17] Wopfner H. & Jin X. C. Pangea Megasequences of Tethyan Gondwana-margin reflect global changes of climate and tectonism in Late Palæozoic and Early Triassic times - a review. Palaeoworld 18, 169–192 (2009).

[b18] Sengör A. M. C. Mid-Mesozoic closure of Permo-Triassic Tethys and its implications. Nature 279, 590–593 (1979).

[b19] Lapierre H. A. . The Tethyan plume: geochemical diversity of middle Permian basalts from the Oman rifted margin. Lithos 74, 167–198 (2004).

[b20] Chauvet F., Dumont T. & Basile C. Structures and timing of Permian rifting in the central Oman Mountains (Saih Hatat). Tectonophysics 475, 563–574 (2009).

[b21] Zhai Q.-G. . SHRIMP zircon U-Pb geochronology, geochemistry and Sr-Nd-Hf isotopic compositions of a mafic dyke swarm in the Qiangtang terrane, northern Tibet and geodynamic implications. Lithos 174, 28–43 (2013).

[b22] Nakazawa K. . The upper Permian and the lower Triassic in Kashmir, India. Mem. Faculty Sci. Kyoto Univ. Ser. Geol. Mineral. 42, 1–106 (1975).

[b23] Shellnutt J. G., Bhat G. M., Brookfield M. E. & Jahn B.-M. No link between the Panjal Traps (Kashmir) and the Late Permian mass extinctions. Geophys. Res. Lett. 38, L19308, 10.1029/2011GL049032 (2011).

[b24] Shellnutt J. G. . Multiple mantle sources of the early Permian Panjal Traps, Kashmir, India. Am. J. Sci. 315, 589–619 (2015).

[b25] Wang M., Li C., Wu Y.-W. & Xie C.-M. Geochronology, geochemistry, Hf isotopic compositions and formation mechanism of radial mafic dykes in northern Tibet. Int. Geol. Rev. 56, 187–205 (2014).

[b26] Stampfli G. M., Hochard C. Vérard C. Wilhem C. & von Raumer J. The formation of Pangea. Tectonophysics 593, 1–19 (2013).

[b27] Campbell I. H. Testing the plume theory. Chem. Geol. 241, 153–176 (2007).

[b28] Ernst R. E. & Buchan K. L. Recognizing mantle plumes in the geological record. Annu. Rev. Earth Pl. Sc. 31, 469–523 (2003).

[b29] Murphy J. B., Strachan R. A., Nance R. D., Parker K. D. & Fowler M. B. Proto-Avalonia: A 1.2–1.0 Ga tectonothermal event and constraints for the evolution of Rodinia. Geology 28, 1071–1074 (2000).

[b30] Stampfli G. M., von Raumer J. & Borel G. D. The Paleozoic Evolution of pre-Variscan Terranes; From peri-Gondwana to the Variscan collision. In: Martinez-Catalan J. R., Hatcher R. E., Arenas R., & Diaz Garcia F. eds Variscan Appalchian Dynamics: the building of the Upper Paleozoic basement*. Geological Society of America Special Paper* 64, 263–280 (2002).

[b31] Stampfli G. M., von Raumer J. & Borel G. D. The distribution of Gondwana-derived terranes in the Early Paleozoic. In: Gutiérrez-Marco J. C., Rábano I. & García-Bellido D. eds The Ordovician of the World*. Cuadernos del Museo Geominero*, Instituto Geológico y Minero de España, Madrid 14, 567–574 (2011).

[b32] Herzberg C. & Asimow P. D. PRIMELT3 MEGA.XLSM software for primary magma calculation: peridotite primary magma MgO contents from the liquidus to the solidus. Geochem. Geophy. Geosy. 16, 563–578 (2015).

[b33] Herzberg C. . Temperatures in ambient mantle and plumes: constraints from basalts, picrites, and komatiites. Geochem. Geophy. Geosy. 8, Q02006, 10.1029/2006GC001390 (2007).

[b34] Bhat M. I., Zainuddin S. M. & Rais A. Panjal Trap chemistry and the birth of Tethys. Geol. Mag. 118, 367–375 (1981).

[b35] Yan Q. . Opening of the Tethys in southwest China and its significance to the breakup of East Gondwanaland in the late Paleozoic: evidence from SHRIMP U-Pb zircon analyses for the Garze ophiolite. Chinese Sci. Bull. 50, 256–264 (2005).

[b36] Fan W., Wang Y., Zhang A., Zhang F. & Zhang Y. Permian arc-back-arc basin development along the Ailaoshan tectonic zone: geochemical, isotopic and geochronological evidence from the Mojiang volcanic rocks, southwest China. Lithos, 119, 553–568 (2010).

[b37] Zhu D.-C. . Presence of Permian extension- and arc-type magmatism in southern Tibet: paleogeographic implications. Geol. Soc. Am. Bull. 122, 979–993 (2010).

[b38] Ali J. R., Aitchison J. C., Chik S. Y. S., Baxter A. T. & Bryan S. E. Paleomagnetic data support Early Permian age for the Arbor volcanics in the lower Siang Valley, NE India: significance for Gondwana break-up models. J. Asian Earth Sci. 50, 105–115 (2012).

[b39] Shellnutt J. G. . Petrogenesis of the flood basalts from the Early Permian Panjal Traps, Kashmir, India: geochemical evidence for shallow melting of the mantle. Lithos 204, 159–171 (2014).

[b40] Herzberg C. & Gazel E. Petrological evidence for secular cooling in mantle plumes. Nature 458, 619–622 (2009). 10.1038/nature07857 19340079

[b41] Coltice N., Phillips B. R., Bertrand H., Ricard Y. & Rey P. Global warming of the mantle at the origin of flood basalts over supercontinents. Geology 35, 391–394 (2007).

[b42] Hole M. J. The generation of continental flood basalts by decompressional melting of internally heated mantle. Geology 43, 311–314 (2015).

[b43] Hole M. J. & Millett J. M. Controls of mantle potential temperature and lithospheric thickness on magmatism in the North Atlantic igneous province. J. Petrol. 57, 417–436 (2016).

[b44] Ali J. R., Fitton J. G. & Herzberg C. Emeishan large igneous province (SW China) and the mantle-plume up-doming hypothesis. J. Geol. Soc. London 167, 953–959 (2010).

[b45] Lin Y.-L. . First evidence of the Cambrian basement in Upper Peninsula of Thailand and its implication for crustal and tectonic evolution of the Sibumasu terrane. Gondwana Res. 24, 1031–1037 (2013).

[b46] Burov E. B. Rheology and strength of the lithosphere. Mar. Petrol. Geol. 28, 1402–1443 (2011).

[b47] López-Gamundí O. R. Glacial-postglacial transition in the late Paleozoic basins of southern South America. In: Martini I. P. ed. Late Glacial and Postglacial Environmental Changes, Quaternary, Carboniferous–Permian and Proterozoic: Oxford, UK, Oxford University Press, p. 147–168 (1997).

[b48] Fielding C. R. . Stratigraphic imprint of the Late Palæozoic ice age in eastern Australia: a record of alternating glacial and nonglacial climate regime. J. Geol. Soc. London 165, 129–140 (2008).

[b49] Jamieson T. F. On the cause of the depression and re-evaluation of the land during the glacial period. Geol. Mag. 9, 400–407 (1882).

[b50] Burov E. B. Ch. 6.03 Plate Rheology and Mechanics A2. In: Schubert G. ed. Treatise on Geophysics (Second Edition). Oxford, Elsevier, 95–152 (2015).

[b51] Johnston A. C. Suppression of earthquakes by large continental ice sheets. Nature 330, 467–469 (1987).

[b52] Braun J. Earth science: glaciers shield mountain tops. Nature 467, 281–282 (2010). 10.1038/467281b 20844527

[b53] Steffen R., Wu P. Steffen H. & Eaton D. W. On the implementation of faults in finite-element glacial isostatic adjustment models. Comput. Geosci. 62, 150–159 (2014).

[b54] Stewart I. S., Sauber J. & Rose J. Glacio-seismotectonics: ice sheets, crustal deformation and seismicity. Quaternary Sci. Rev. 19, 1367–1389 (2000).

[b55] Sibson R. H., Frictional Constraints on thrust, wrench and normal faults. Nature 249, 542–544 (1974).

[b56] Fan J.-J., Li C. Wang M., Xie C.-M. & Xu W. Features, provenance, and tectonic significance of Carboniferous–Permian glacial marine diamictites in the Southern Qiangtang–Baoshan block, Tibetan Plateau. Gondwana Res. 28, 1530–1542 (2015).

[b57] Torsvik T. H., Robin L. & Cocks M. Earth geography from 400 to 250 Ma: a palaeomagnetic, faunal and facies review. J. Geol. Soc. London 161, 555–572 (2004).

[b58] Veevers J. J. Gondwanaland from 650–500 Ma assembly through 320 Ma merger in Pangea to 185–100 Ma breakup: supercontinental tectonics via stratigraphy and radiometric dating. Earth-Sci. Rev. 68, 1–132 (2004).

[b59] Ali J., Cheung M., Aitchison. J. C. & Sun Y. Paleomagnetic re-investigation of Early Permian rift basalts from the Baoshan Block, SW China: constraints on the site-of-origin of the Gondwana-derived eastern Cimmerian terranes. Geophys. J. Int. 193, 650–663 (2013).

[b60] Sengör A. M. C., Altiner D., Ustaomer T. & Hsu K. J. Origin and assembly of the Tethyside orogenic collage at the expense of Gondwana-land. In: Audley-Charles M. G. & Mallam A. eds Geological Society, London, Special Publication 37, 119–181 (1988).

[b61] Muttoni G. . Early Permian Pangea ‘B’ to Late Permian Pangea ‘A’. Earth Planet. Sc. Lett. 215, 379–394 (2003).

[b62] Domeier M. . Support for an “A-type” Pangea reconstruction from high-fidelity Late Permian and Early to Middle Triassic paleomagnetic data from Argentina. J. Geophys. Res. 116, B12114 (2011).

[b63] Metcalfe I. Gondwana dispersion and Asian accretion: Tectonic and palaeogeographic evolution of eastern Tethys. J. Asian Earth Sci. 66, 1–33 (2013).

[b64] Seton M. . Global continental and ocean basin reconstructions since 200 Ma. Earth-Sci. Rev. 113, 212–270 (2012).

[b65] Torsvik T. H. & Cocks L. R. M. Gondwana from top to base in space and time. Gondwana Res. 24, 999–1030 (2013).

[b66] Zanchetta S. . The record of the Late Palæozoic active margin of the Palaeotethys in NE Iran: Constraints on the Cimmerian orogeny. Gondwana Res. 24, 1237–1266 (2013).

[b67] Wang S., Mo Y., Wang C. & WYe P. Paleotethyan evolution of the Indochina Block as deduced from granites in northern Laos. Gondwana Res. 10.1016/j.gr.2015.11.011 (2016).

[b68] Stojanovic D., Aitchison J. C., Ali J. R., Ahmad T. & Dar R. A. Paleomagnetic investigation of the Early Permian Panjal Traps of NW India; regional tectonic implications. J. Asian Earth Sci. 115, 114–123 (2016).

[b69] Ali J. R. & Aitchison J. C. Greater India. Earth-Sci. Rev. 72, 169–188 (2005).

[b70] Xu W., Dong Y. Zhang X., Deng M. & Zhang L. Petrogenesis of high-Ti mafic dykes from Southern Qiangtang, Tibet: Implications for a ca. 290 Ma large igneous province related to the early Permian rifting of Gondwana. Gondwana Res. 10,1016/j.gr.2015.07.016 (2016).

[b71] Metcalfe I. Tectonic framework and Phanerozoic evolution of Sundaland. Gondwana Res. 19, 3–21 (2011).

[b72] Zhai Q.-g. . Triassic arc magmatism in the Qiangtang area, northern Tibet: Zircon U–Pb ages, geochemical and Sr–Nd–Hf isotopic characteristics, and tectonic implications. J. Asian Earth Sci. 63, 162–178 (2013).

[b73] Bussert R. & Schrank E. Palynological evidence for a latest Carboniferous-Early Permian glaciation in Northern Ethiopia. J. Afr. Earth Sci. 49, 201–210 (2007).

[b74] Catuneanu O. . The Karoo basins of south-central Africa. J. Afr. Earth Sci. 43, 211–253 (2005).

[b75] Eyles N., Mory A. J. & Backhouse J. Carboniferous–Permian palynostratigraphy of west Australian marine rift basins: resolving tectonic and eustatic controls during Gondwanan glaciations. Palaeogeogr. Palaeoecl. 184, 305–319 (2002).

[b76] Henry L. C., Isbell J. L. & Limarino C. O. The late Paleozoic El Imperial Formation, western Argentina: Glacial to post-glacial transition and stratigraphic correlations with arc-related basins in southwestern Gondwana. Gondwana Res. 25, 1380–1395 (2014).

[b77] Limarino C. O. . A paleoclimatic review of southern South America during the late Paleozoic: A record from icehouse to extreme greenhouse conditions. Gondwana Res. 25, 1396–1421 (2014).

[b78] Pazos P. J. The Late Carboniferous Glacial to Postglacial Transition: Facies and Sequence Stratigraphy, Western Paganzo Basin, Argentina. Gondwana Res. 5, 467–487 (2002).

[b79] Fielding C. R. . Stratigraphic imprint of the Late Paleozoic ice age in eastern Australia: a record of alternating glacial nonglacial climate regime. J. Geol. Soc. London 165, 129–140 (2008).

[b80] Visser J. N. J. Post-glacial Permian stratigraphy and geography of southern and central Africa: boundary conditions for climatic modelling. Palaeogeogr. Palaeocl. 118, 213–243 (1995).

[b81] Wopfner H. The early Permian deglaciation event between East Africa and northwestern Australia. J. Afr. Earth Sci. 29, 77–90 (1999).

[b82] Chumakov N. M. & Zharkov M. A. Climate during Permian–Triassic Biosphere Reorganizations, Article 1: Climate of the Early Permian. Stratigr. Geol. Correl. 10, 586–602 (2002).

[b83] Golonka J. Late Triassic and Early Jurassic palaeogeography of the world. Palaeogeogr. Palaeocl. 244, 297–307 (2007).

[b84] Golonka J. & Ford D. Pangean (Late Carboniferous–Middle Jurassic) paleoenvironment and lithofacies. Palaeogeogr. Palaeocl. 161, 1–34 (2000).

[b85] Isbell J. . Glacial paradoxes during the late Paleozoic ice age: Evaluating the equilibrium line altitude as a control on glaciation. Gondwana Res. 22, 1–19 (2012).

