# Supplementary material for: The initial break-up of Pangæa elicited by Late Palæozoic deglaciation
Source: Sci Rep. 2016 Aug 11;6:31442. doi: 10.1038/srep31442 (PMC4980595; doi:10.1038/srep31442)
Supplement: Supplementary Information [file srep31442-s1.doc]

# Supplementary Information

# The initial break-up of Pangæa elicited by Late Palæozoic deglaciation

Meng-Wan Yeh^1,2^, and J. Gregory Shellnutt^1*^

*^1^Department of Earth Sciences, National Taiwan Normal University, 88 Tingzhou Road Section 4, Taipei 116, Taiwan*

*^2^Center for General Education, National Taiwan Normal University, 162 Heping East Road Section 1, Taipei 106, Taiwan*

*Correspondence and requests for materials should be addressed to J.G.S. (email:jgshelln@ntnu.edu.tw)

**Supplementary Dataset S1**

**Dataset S1.** The distribution of Permo-Carboniferous sedimentary formation/units with glacial deposits reconstructed via tracing the stratigraphic columns within major basins of the Gondwana terranes

| **Region** | **Location** | **Age range** | **Formation/Unit** | **References** |
| --- | --- | --- | --- | --- |
| South America | Parana basin | Late Visean- Early Bashkirian (347 Ma - 315 Ma) | Irarare group | 77 and references within |
| South America | Rio Blanco basin | Late Visean- Early Bashkirian (347 Ma - 315 Ma) | Cortaderas formation, Rio del Penon formation | 77 and references within |
| South America | Western Paganzo basin | Late Carboniferous | Guandacol formation, Loma de Los Piojos formation | 78, 77 and references within |
| South America | Calingasta-Uspallata basin | Late Visean- Early Bashkirian (347 Ma - 315 Ma) | Hoyada Verde formation | 77 and references within |
| South America | San Rafael basin | Late Visean- Early Bashkirian (347 Ma - 315 Ma) | El Imperial formation | 77 and references within |
| South America | Tepuel basin | Late Visean- Early Bashkirian (347 Ma - 315 Ma) | Pampa de Tepuel | 76 |
| Arabian peninsula | South Yemen and South Oman basins | Stephanian (304 Ma - 292 Ma) | Al Khlata Formation | 17 and references within |
| North Africa | Ethiopia | Late Carboniferous - Early Permian | Edaga Arbi Glacials | 73 |
| Eastern Africa | Ruhuhu basins | Stephanian - Sakamarian (304 Ma - 292 Ma) | Indusi formation | 17 and references within |
| Madagascar | Morondava basin | Late Carboniferous - Early Permian | Pangea Megasequence | 17 and references within |
| South Africa | Kalahari basin | Late Permiam | Tlhabala formation | 80 and references within |
| South Africa | Namibia | Late Permiam | Pangea Megasequence | 80 and references within |
| South Africa | Angola | Late Permiam | Upper Lukuga beds | 80 and references within |
| South Africa | Zimbabwe | Late Permiam | Hatambulo formation | 80 and references within |
| South Africa | Zambia | Late Permiam | Madumabisa mudstone | 80 and references within |
| South Africa | Mozambique | Late Permiam | Hatambulo formation | 80 and references within |
| South Africa | Karoo basins | Permo-Carboniferous to Mid-Triassic unconformity | Idusi Formation Lisimba member | 17 and references within |
| South Africa | Karoo basins | Late Carboniferous (300 Ma - 290 Ma) | Dwyka group | 80 and references within, 74 |
| India | Kashmir | Assalian (299 Ma - 295 Ma) | Mid Agglomeratic slate | 17 and references within |
| India | Sikkim, Arunachal Pradesh | Assalian (299 Ma - 295 Ma) | Lower Rangit Pep. Slate | 17 and references within |
| Lhasa block | Poindo area | Assalian - Sakmarian (299 Ma - 290 Ma) | Poindo group | 17 and references within |
| Lhasa block | Qomolangma | Assalian - Sakmarian (299 Ma - 291 Ma) | Jilong group | 17 and references within |
| Qiangtang block | Orba lake and Rutog | Assalian - Sakmarian (299 Ma - 292 Ma) | Cameng Formation | 17 and references within |
| Tengchong block | Kongshue village | Stephanian (304 Ma - 299 Ma) | Kongshuhe Formaiton | 17 and references within |
| Qiangtang -Baoshan block |  | Carboniferous - Permian | Zhanjin Formation | 56 |
| Australia | Carnarvon basin | Carboniferous- Late Permian | Lyons group | 75 and references within |
| Australia | Perth basin | Carboniferous- Late Permian | Cullerns Diamictite, Nangetty formation | 75 and references within |
| Australia | Gunbarrel basin | Stephanian-Assalian (304 Ma - 295 Ma) | Paterson formation | 75 and references within |
| Australia | Collie basin | Stephanian (304 Ma - 299 Ma) | Shotts formation | 75 and references within |
| Australia | Canning basin | Stephanian-Assalian (304 Ma - 295 Ma) | Hoya formation, Reeves formation | 17, 75 and references within |
| Australia | Bonaparte Gulf basin | Assalian - Sakmarian (299 Ma - 290 Ma) | Kulshill group | 17 and references within |
| Australia | Arafura Basin | Carbo Stephanian - Sakamarian stage (304 Ma - 292 Ma) | Pangea Megasequence | 17 and references within |
| Australia | Bowen Basin | Permian | Peawaddy formation, Cattle Creek formation | 79 and references within |
| Australia | Gunnedah Basin | Carboniferous – Late Permian | Watermark, Porcupine group, Goonbr formation | 79 and references within |
| Australia | Galilee Basin | Permian | Betts Creek Beds, Boonderee Beds | 79 and references within |
| Australia | Cooper Basin | Assalian - Sakmarian (299 Ma - 290 Ma) | Tirrawarra Sandstone | 79 and references within |
| Australia | Sydney Basin | Permian | Wilton and Erins Vale formation, Pebbley Beach Formation, Wasp Head Formation, Branxton Formation, Lochinvar Formation | 79 and references within |
| Australia | Southern New England Fold Belt | Carboniferous – Late Permian | Currabubula Formation, Seaham Formation, Beckers Formation, Billy Brook Formation, Branzton Formation | 79 and references within |

**Supplementary Dataset S2**

**Dataset S2**. Primary melt compositions and mantle potential temperatures of Early Permian basalts from Oman, Kashmir and Qiangtang

| Sample | OM123 | AFM | AFM | OM123* | AFM | AFM | OM13 | AFM | AFM | OM13* | AFM | AFM | OM109 | AFM | AFM |
| --- | --- | --- | --- | --- | --- | --- | --- | --- | --- | --- | --- | --- | --- | --- | --- |
| Region | Oman |  |  | Oman |  |  | Oman |  |  | Oman |  |  | Oman |  |  |
| SiO_2_ (wt.%) | 46.9 | 48.29 | 48.30 | 48.93 | 48.29 | 48.30 | 51.50 | 52.56 | 52.92 | 53.72 | 52.56 | 52.92 | 47.81 | 46.92 | 47.08 |
| TiO_2_ | 0.76 | 0.71 | 0.71 | 0.79 | 0.70 | 0.71 | 0.90 | 0.84 | 0.87 | 0.94 | 0.84 | 0.87 | 1.19 | 1.00 | 1.03 |
| Al_2_O_3_ | 15.3 | 14.18 | 14.20 | 15.96 | 14.18 | 14.20 | 13.75 | 12.82 | 13.21 | 14.34 | 12.81 | 13.19 | 17.02 | 14.23 | 14.62 |
| Fe_2_O_3_ | 9.04 | 0.35 | 0.71 | 9.43 | 0.35 | 0.70 | 7.78 | 0.42 | 0.86 | 8.12 | 0.42 | 0.86 | 10.32 | 0.50 | 1.02 |
| FeO |  | 8.61 | 8.24 |  | 8.62 | 8.25 |  | 7.48 | 6.94 |  | 7.49 | 6.95 |  | 9.24 | 8.68 |
| FeOt | 8.13 |  |  | 8.49 |  |  | 7.00 |  |  | 7.31 |  |  | 9.29 |  |  |
| MnO | 0.14 | 0.15 | 0.15 | 0.15 | 0.15 | 0.15 | 0.14 | 0.15 | 0.15 | 0.15 | 0.16 | 0.16 | 0.18 | 0.18 | 0.18 |
| MgO | 7.75 | 12.88 | 12.85 | 8.09 | 12.88 | 12.85 | 7.24 | 12.13 | 11.04 | 7.55 | 12.13 | 11.06 | 8.45 | 15.32 | 14.42 |
| CaO | 13.05 | 12.13 | 12.15 | 13.61 | 12.12 | 12.14 | 13.45 | 12.57 | 12.95 | 14.03 | 12.57 | 12.94 | 11.73 | 9.85 | 10.11 |
| Na_2_O | 2.78 | 2.57 | 2.58 | 2.90 | 2.57 | 2.58 | 0.98 | 0.91 | 0.94 | 1.02 | 0.91 | 0.94 | 3.06 | 2.56 | 2.63 |
| K_2_O | 0.06 | 0.06 | 0.06 | 0.06 | 0.05 | 0.05 | 0.02 | 0.02 | 0.02 | 0.02 | 0.02 | 0.02 | 0.12 | 0.10 | 0.10 |
| P_2_O_5_ | 0.08 | 0.07 | 0.07 | 0.08 | 0.07 | 0.07 | 0.10 | 0.09 | 0.10 | 0.10 | 0.09 | 0.09 | 0.14 | 0.12 | 0.12 |
| Pressure (bars) |  | 1 | 1 |  | 1 | 1 |  | 1 | 1 |  | 1 | 1 |  | 1 | 1 |
| FeO (source) |  | 8.62 | 8.27 |  | 8.63 | 8.28 |  | 8.47 | 8.42 |  | 8.48 | 8.42 |  | 8.15 | 8.02 |
| MgO (source) |  | 38.12 | 38.12 |  | 38.12 | 38.12 |  | 38.12 | 38.12 |  | 38.12 | 38.12 |  | 38.12 | 38.12 |
| Fe_2_O_3_/TiO_2_ |  | 0.5 | 1.0 |  | 0.5 | 1.0 |  | 0.5 | 1.0 |  | 0.5 | 1.0 |  | 0.5 | 1.0 |
| % ol addition |  | 12.9 | 12.7 |  | 12.9 | 12.7 |  | 12.2 | 9.1 |  | 12.2 | 9.2 |  | 19.1 | 16.3 |
| Melt Fraction |  | 0.06 | 0.06 |  | 0.06 | 0.06 |  | 0.28 | 0.27 |  | 0.28 | 0.27 |  | 0.10 | 0.09 |
| Temperature (^o^C) |  | 1300 | 1300 |  | 1300 | 1300 |  | 1260 | 1240 |  | 1360 | 1240 |  | 1360 | 1340 |
| T_P_ (^o^C) |  | 1380 | 1380 |  | 1380 | 1380 |  | 1360 | 1330 |  | 1360 | 1330 |  | 1440 | 1420 |

FeOt = Fe_2_O_3_t * 0.8998. * = duplicate analysis. AFM = accumulated fractional melting composition. The model compositions are normalized to 100% for the PRIMELT3 calculation^19,21,24,37,39^.

| Sample | OM117 | AFM | AFM | OM117* | AFM | AFM | PJ2-003 | AFM | AFM | PJ4-006 | AFM | AFM | E713 | AFM | AFM |
| --- | --- | --- | --- | --- | --- | --- | --- | --- | --- | --- | --- | --- | --- | --- | --- |
| Region | Oman |  |  | Oman |  |  | Kashmir |  |  | Kashmir |  |  | Tibet |  |  |
| SiO_2_ (wt.%) | 47.00 | 47.78 | 48.10 | 48.86 | 47.78 | 48.10 | 51.23 | 50.62 | 50.90 | 52.14 | 51.97 | 52.33 | 48.02 | 48.05 | 48.47 |
| TiO_2_ | 1.39 | 1.22 | 1.27 | 1.45 | 1.22 | 1.28 | 0.76 | 0.64 | 0.66 | 0.98 | 0.87 | 0.90 | 1.74 | 1.48 | 1.57 |
| Al_2_O_3_ | 15.45 | 13.47 | 14.10 | 16.06 | 13.48 | 14.11 | 14.70 | 12.30 | 12.63 | 12.37 | 10.90 | 11.25 | 14.49 | 12.28 | 12.99 |
| Fe_2_O_3_ | 9.10 | 0.60 | 1.27 | 9.46 | 0.61 | 1.27 | 9.04 | 0.32 | 0.65 | 8.69 | 0.43 | 0.89 | 10.10 | 0.74 | 1.56 |
| FeO |  | 8.56 | 7.81 |  | 8.55 | 7.80 |  | 9.01 | 8.62 |  | 8.19 | 7.67 |  | 9.04 | 8.19 |
| FeOt | 8.19 |  |  | 8.51 |  |  | 8.35 |  |  | 7.82 |  |  | 9.09 |  |  |
| MnO | 0.15 | 0.16 | 0.16 | 0.16 | 0.17 | 0.17 | 0.15 | 0.16 | 0.16 | 0.15 | 0.16 | 0.16 | 0.15 | 0.16 | 0.16 |
| MgO | 6.60 | 13.78 | 12.18 | 6.86 | 13.76 | 12.16 | 7.50 | 15.44 | 14.55 | 7.37 | 13.65 | 12.54 | 8.26 | 15.78 | 13.88 |
| CaO | 12.80 | 11.21 | 11.72 | 13.31 | 11.22 | 11.73 | 12.24 | 10.28 | 10.56 | 11.90 | 10.52 | 10.86 | 11.74 | 9.99 | 10.56 |
| Na_2_O | 3.46 | 3.01 | 3.16 | 3.60 | 3.02 | 3.16 | 1.33 | 1.11 | 1.14 | 3.25 | 2.86 | 2.95 | 2.27 | 1.92 | 2.03 |
| K_2_O | 0.04 | 0.04 | 0.04 | 0.04 | 0.03 | 0.04 | 0.07 | 0.06 | 0.06 | 0.43 | 0.38 | 0.39 | 0.47 | 0.40 | 0.42 |
| P_2_O_5_ | 0.20 | 0.17 | 0.18 | 0.21 | 0.18 | 0.18 | 0.08 | 0.07 | 0.07 | 0.07 | 0.06 | 0.06 | 0.20 | 0.17 | 0.18 |
| Pressure (bars) |  | 1 | 1 |  | 1 | 1 |  | 1 | 1 |  | 1 | 1 |  | 1 | 1 |
| FeO (source) |  | 8.02 | 8.02 |  | 8.02 | 8.02 |  | 8.54 | 8.53 |  | 8.47 | 8.43 |  | 8.26 | 8.19 |
| MgO (source) |  | 38.12 | 38.12 |  | 38.12 | 38.12 |  | 38.12 | 38.12 |  | 38.12 | 38.12 |  | 38.12 | 38.12 |
| Fe_2_O_3_/TiO_2_ |  | 0.5 | 1.0 |  | 0.5 | 1.0 |  | 0.5 | 1.0 |  | 0.5 | 1.0 |  | 0.5 | 1.0 |
| % ol addition |  | 18.6 | 14.0 |  | 18.6 | 13.9 |  | 21.8 | 19.0 |  | 16.4 | 13.1 |  | 20.4 | 14.5 |
| Melt Fraction |  | 0.04 | 0.02 |  | 0.04 | 0.02 |  | 0.30 | 0.29 |  | 0.28 | 0.27 |  | 0.24 | 0.22 |
| Temperature (^o^C) |  | 1330 | 1300 |  | 1330 | 1300 |  | 1340 | 1320 |  | 1330 | 1300 |  | 1360 | 1330 |
| T_P_ (^o^C) |  | 1400 | 1360 |  | 1400 | 1360 |  | 1450 | 1420 |  | 1400 | 1370 |  | 1460 | 1400 |

| Sample | NX1-1 | AFM | AFM | MG706 | AFM | AFM |
| --- | --- | --- | --- | --- | --- | --- |
| Region | Tibet |  |  | Tibet |  |  |
| SiO_2_ (wt.%) | 48.95 | 47.37 | 47.37 | 52.11 | 52.82 | 53.38 |
| TiO_2_ | 0.89 | 0.70 | 0.70 | 1.59 | 1.52 | 1.59 |
| Al_2_O_3_ | 17.11 | 13.44 | 13.42 | 11.03 | 10.54 | 11.04 |
| Fe_2_O_3_ | 10.07 | 0.35 | 0.70 | 8.51 | 0.76 | 1.59 |
| FeO |  | 9.55 | 9.12 |  | 7.43 | 6.54 |
| FeOt | 9.06 |  |  | 7.66 |  |  |
| MnO | 0.18 | 0.19 | 0.19 | 0.16 | 0.17 | 0.16 |
| MgO | 6.46 | 15.52 | 15.64 | 8.80 | 12.03 | 10.28 |
| CaO | 12.44 | 9.82 | 9.81 | 11.90 | 11.39 | 11.92 |
| Na_2_O | 2.55 | 2.00 | 2.00 | 2.49 | 2.38 | 2.49 |
| K_2_O | 1.15 | 0.90 | 0.90 | 0.83 | 0.79 | 0.83 |
| P_2_O_5_ | 0.19 | 0.15 | 0.15 | 0.17 | 0.16 | 0.17 |
| Pressure (bars) |  | 1 | 1 |  | 1 | 1 |
| FeO (source) |  | 8.58 | 8.16 |  | 8.44 | 8.34 |
| MgO (source) |  | 38.12 | 38.12 |  | 38.12 | 38.12 |
| Fe_2_O_3_/TiO_2_ |  | 0.5 | 1.0 |  | 0.5 | 1.0 |
| % ol addition |  | 25.4 | 25.5 |  | 7.8 | 3.1 |
| Melt Fraction |  | 0.17 | 0.18 |  | 0.27 | 0.25 |
| Temperature (^o^C) |  | 1360 | 1370 |  | 1290 | 1250 |
| T_P_ (^o^C) |  | 1450 | 1450 |  | 1360 | 1310 |
